# Psychometric Properties of the Italian Version of the Burnout Assessment Tool (BAT)

**DOI:** 10.3390/ijerph18189469

**Published:** 2021-09-08

**Authors:** Chiara Consiglio, Greta Mazzetti, Wilmar B. Schaufeli

**Affiliations:** 1 Department of Psychology, Faculty of Medicine & Psychology Sapienza, University of Rome, Via dei Marsi 78, 00185 Rome, Italy; 2 Department of Education Studies “G. M. Bertin”—Alma Mater Studiorum, Università di Bologna, 40126 Bologna, Italy; greta.mazzetti3@unibo.it; 3 Research Unit Occupational & Organizational Psychology and Professional Learning, KU Leuven, 3000 Leuven, Belgium; wilmar.schaufeli@kuleuven.be or; 4 Department of Psychology, Utrecht University, P.O. Box 80.140, 3508 TC Utrecht, The Netherlands

**Keywords:** BAT Burnout Assessment Tool, burnout, MBI, MTMM, psychometric properties

## Abstract

The most popular instrument to measure burnout is the Maslach Burnout Inventory (MBI). Recently, to overcome some of the limitations of the MBI, a new instrument has been proposed, namely the Burnout Assessment Tool. The purpose of this study is to examine the psychometric properties of the Italian version of the BAT. This tool is comprised of a set of four core dimensions (BAT-C; i.e., exhaustion, mental distance, cognitive and emotional impairment) and two secondary symptom dimensions (BAT-S; i.e., psychological and psychosomatic complaints). Data were collected on a sample of 738 participants from heterogeneous sectors and professional roles. In the sample women were slightly overrepresented (52.9%), the participants had a mean age of 41.57 years (SD = 10.51) and a mean organizational tenure of 9.65 years (SD = 8.50). The reliability and factorial structure of the BAT-C and BAT-S, together with the convergent and discriminant validity of BAT-C and MBI were explored, as well as the incremental validity to the BAT-C, over and beyond the MBI. Our results confirmed the factorial validity of a two-factor second-order factor model (BAT-C and BAT-S) represented by 4 first-order factors in the case of BAT-C and 2 first-order factors for BAT-S. Results also attested that BAT-C explains additional variance of the BAT-S, above and beyond what is explained by the MBI-GS. All in all, this study provided evidence that the Italian version of BAT represents a reliable and valid tool for measuring burnout in the work context.

## 1. Introduction

Burnout is a very well-studied concept that refers to individuals’ response to chronic, work-related stress [1]. Over the past 45 years, burnout has inspired thousands of articles and books, drawing the attention of both scholars and practitioners perhaps more than any other construct [2,3]. According to Google Scholar to date, over 1,200,000 publications have been written on burnout and, among these, approximately 12,000 are included in peer-reviewed journals [4].

Even though the burnout phenomenon was originally studied among health care professionals, and conceived as resulting from emotionally-demanding work interactions with patients/recipients, it was later redefined as a more general phenomenon that may occur across different working contexts, as a result of a wide range of job demands [5,6,7]. Recently, the World Health Organization has included burnout as an “occupational phenomenon” influencing health (see the 11th revision of the International Classification of Diseases) [8]. Thus, burnout is currently classified as a condition which may affect any employee, regardless of their job role, organizational sector, or country [2].

Burnout represents an occupational health problem, which requires growing awareness, especially due to its negative consequences for individuals and organizations. Physical and psychological ill-health have been extensively associated to burnout, including cardiovascular and metabolic disease problems [9,10,11], musculoskeletal disorders [12,13], need for recovery [14] and depressive and sleep symptoms [15,16,17,18]. Negative occupational outcomes associated with burnout include sickness absences [19,20,21], poor job performance [22,23], turnover intentions [24,25,26] and worker and patient negative safety outcomes [27,28]. Moreover, burnout is often considered a social problem, especially in welfare states, where national social health systems cover sickness absence and work-related health problems [2,24]. Therefore, in order to assess and prevent this phenomenon, it is crucial to have reliable and valid tools and shared criteria to measure burnout risk.

Despite great interest from different stakeholders (scholars, organizations, policy makers, institutions), and its relevant direct and indirect consequences, a recent comparative review conducted by Eurofound [29] underlined that most burnout evidence is based on small scale occupational studies, which make it difficult to identify, contrast and compare burnout prevalence and burnout risk levels across countries. Moreover, in some countries, burnout is viewed as a work-related syndrome (which can be assessed based on self-reported measures), while in others (such as the Netherlands, Denmark, Estonia, France, Hungary, Latvia, Netherlands, Portugal, Slovakia and Sweden), burnout is recognized as an occupational disease (requiring a specific medical diagnosis) [30]. Therefore, burnout data sources and diagnostic measures are hardly comparable [29]. This evidence strongly suggests the need to harmonize assessment tools and criteria for burnout risk levels.

The most recognized definition of the burnout concept refers to Maslach’s seminal work, which describes it as a work-related stress syndrome composed of three dimensions; namely exhaustion, depersonalization and reduced professional efficacy [31].

Exhaustion refers to the feeling of having drained one’s psychological and physical resources, depersonalization corresponds to the detached and indifferent attitude towards the recipient (subsequently named as cynicism, which describes a detached and indifferent response towards work) while (reduced) professional efficacy (originally known as personal accomplishment) represents the perception of the employee’s efficacy, competence and productivity [31]. From this conceptualization, Maslach operationalized the Maslach Burnout Inventory (MBI, [6]) in its different versions, namely the Health Services Survey (HSS), the Educational Survey (ES) and the General Survey versions (GS). Despite the existence of other tools to measure burnout, such as the Copenhagen Burnout Inventory (CBI, [32]), The Oldenburg Burnout Inventory (OLBI, [33]) and the Shirom-Melamed Burnout Measure (SMBM, [34]), the MBI is undoubtedly the most popular instrument to assess burnout, used in most published research (about 88%) [35]. Consequently, burnout has often been defined as “the concept measured by the MBI”, and this reciprocal dependence of the concept and instrument has probably hindered the development and spread of other tools and, therefore, a better understanding of the burnout phenomenon.

Despite its remarkable success, the MBI has been criticized for conceptual, methodological, and practical reasons [36]. From a conceptual point of view, the MBI was originally developed “inductively” from interviews conducted with health professionals [31], without a clear conceptual model accounting for its three dimensions. In fact, the presence of these underlying dimensions emerged from a factor-analysis. In particular, professional efficacy has often been considered as being independent from cynicism and exhaustion, and acting as a consequence of burnout, rather than a constitutive burnout dimension. In line with this view, in 2005 Schaufeli and Taris [7] recognized exhaustion and cynicism as the two core burnout dimensions, corresponding to the “inability” (exhaustion) and unwillingness (cynicism) to work, referring to the energetic and motivational component of burnout, respectively. The conceptualization of burnout has advanced from being merely attributable to human service workers, to a condition which can occur across all work contexts (e.g., [37]). Accordingly, some authors have questioned the overlap between depersonalization and cynicism entailed in the MBI, arguing the necessity of considering them as conceptually and empirically distinct dimensions [38,39].

Other limitations of the MBI refer to psychometrical and methodological aspects: in particular, issues related to factor validity [40,41,42], and the presence of a negative dimension measured with positive items (i.e., reduced professional efficacy). Some studies have raised doubts about the factorial invariance of the MBI across countries, as it has emerged as being problematic in several cases [43]. Moreover, reliability problems related to item wording emerged, particularly for Personal accomplishment and Depersonalization [44]. The latter also showed a non-normal and positively biased distribution [32], because of the negative reactions they generated among respondents. 

Finally, other criticisms regarding the MBI involve the practical use of the instrument. For example, according to the MBI test manual [45], it produces three separate scores which should not be combined into a single burnout score. In fact, the MBI was created mainly for research purposes and not for individual assessment, therefore this approach may have produced some difficulties in creating burnout cut-off scores and in defining burnout risk levels [36].

To overcome these limitations, a new tool has been recently developed, namely the Burnout Assessment Tool (BAT, [36,46]), aimed at proposing a new and unique conceptualization of the burnout phenomenon and a methodologically sound tool able to assess burnout as a whole.

The BAT was developed by combining a deductive and an inductive approach: specifically, the research team reconceptualized the burnout concept starting from the two basic universally-recognized burnout components [7]: (1) the energetic dimension (namely the feeling of being exhausted, drained, and worn out by the work) corresponding to the inability to work (labeled Exhaustion); and (2) the motivational dimension (the feeling of detachment, disillusionment and aversion towards work) corresponding to the unwillingness to work (labeled “mental distance”). Subsequently, 49 in-depth interviews of professionals working with burnout workers (e.g., psychologists, occupational physicians, and general practitioners) were conducted to identify recurrent burnout symptoms. These symptoms were then categorized by means of content analysis, which gave rise to seven dimensions, which, in turn, were grouped into primary and secondary burnout symptoms. Based on the evidence that all professionals described both cognitive and emotional impairment symptoms [36], these two dimensions were added to exhaustion and mental distance, as core burnout symptoms. The former is defined as the difficulty to adequately control cognitive processes (such as low attention, concentration, memory) to do the work, and the latter as difficulties in regulating emotions (negative emotions, irritation, unmotivated emotional reactions). These four core symptoms are often associated with other recurrent symptoms: (1) a variety of non-specific psychological symptoms (e.g., anxiety, and sleep disturbances); (2) psychosomatic symptoms (e.g., chest pain, stomachache, and palpitations); and (3) a depressive mood (e.g., feelings of sadness and hopelessness). Therefore, these three dimensions were defined as secondary burnout symptoms [46].

In the second phase, in order to identify the items of the four scales, nine existing burnout questionnaires including a total of 50 burnout scales, were analyzed in detail. Depressive mood was not included in the BAT since there are extensively validated depression scales available, therefore this dimension was comprised only in the conceptual model [36].

The third phase consisted of the development of the questionnaire, comprising 33 items, 23 referring to the four core symptoms, and 10 referring to the secondary symptoms. The first validation study of the BAT is presented in the BAT manual [46], which showed its good psychometric properties based two large, representative samples of the Dutch and Flemish working population. Regarding the core symptoms, the bi-factor model with four first-order factors (exhaustion, mental distance, cognitive and emotional impairment) and one general, second-order, burnout factor fitted the data well. Furthermore, all four scales showed good reliability in terms of internal consistency and stability across time. Convergent validity between the BAT, the MBI and the OLBI was also found; as well as divergent validity with workaholism and boredom.

A second study was performed on a representative sample of Belgian workers [36]. The BAT showed adequate psychometric properties: prominent levels of reliability (>0.81) and a factorial structure with a second order factor representing the four core symptoms. However, in this case, psychological and psychosomatic complaints collapsed into a single factor. Moreover, high convergent validity with two burnout measures (the MBI and the OLBI) and adequate discriminant validity (with engagement and workaholism) was largely supported. In general, the choice of performing a second-order model is in line with the perspective that burnout is the underlying condition (or syndrome) that presents itself through the four (first order factors) symptoms. This perspective is also supported by the results of the Rasch model, which attested that the BAT is one-dimensional; that is, that the four core-dimensions of burnout can be added to constitute a single, composite burnout-score [4].

The core burnout symptoms are particularly relevant to the empirical exploration of burnout, as they represent its most recurrent and pervasive manifestation among workers [47]. On the other hand, secondary symptoms are—by definition—non-specific as they may also develop as a result of other mental (e.g., mood disorder, anxiety disorder) and physical (e.g., CFS, hypo/hyper-thymia) disorders. Therefore, it could be questioned to what extent the core-burnout symptoms co-occur with (‘predict’ in statistical terms, when assessing the predictive validity) the secondary symptoms.

Finally, De Beer and colleagues [48], conducted a study in which the cross-national measurement invariance of the BAT was successfully demonstrated across representative samples from seven different countries (i.e., Belgium, The Netherlands, Germany, Austria, Finland, Ireland, and Japan). This study demonstrated the possibility of using the BAT to assess and compare burnout levels across countries. Moreover, results showed that in Japan, burnout is more prevalent compared to all European countries.

To date, the BAT has been translated into 24 languages. So far, validation studies have appeared around the Dutch [36], Japanese [49], Brazilian [50], and Ecuadorian versions [51], while studies in other countries are in progress.

This study aims to evaluate the psychometric properties of the Italian BAT, which includes both the core and secondary burnout symptoms, using the following steps:

(a) We assessed the factor structure of the core dimensions (BAT-C) and the secondary symptoms (BAT-S) of burnout by using an Exploratory Factor Analysis (EFA);
(b) The reliability of the scales was then evaluated in terms of internal consistency, through the Cronbach’s alpha coefficient;
(c) The factor structure that emerged from the EFA was validated by using Confirmatory Factor Analysis (CFA);
(d) To assess the convergent and discriminant validity (of the BAT vis-à-vis other burnout instruments (i.e., MBI), four alternative MTMM models were compared;
(e) A hierarchical regression was performed to evaluate the predictive and incremental validity of the BAT-C above and beyond the MBI-GS;
(f) The descriptive results obtained on the Italian sample were compared with data obtained across seven nationally representative samples, as reported in De Beer (2020) [48].

## 2. Materials and Methods

### 2.1. Translation

The Italian version of the BAT was obtained by performing a conventional translation and back-translation procedure [52]. The English version of the BAT was translated into Italian by three researchers who worked for at least 10 years as academics or organizational psychologists (two of them are authors of the current paper). Then, a qualified native-speaker translator with no formal knowledge of the original scale translated them back into English. The original English and the back-translated versions were reviewed to highlight any inconsistencies and harmonize them. This led to the Italian version of the BAT scale reported in Appendix A.

### 2.2. Participants

In order to explore the psychometric properties of the Italian version of the BAT, data were collected on a sample of 738 participants. The current study was part of a research project concerning work-related psychosocial risk assessments across several organizations belonging to different occupational sectors. The occupational sectors involved in the study are reported in Table 1, along with a full description of respondents’ characteristics.

Participants received an email containing an anonymous link allowing them to fill in an online questionnaire on an occupational health website. The first page of the questionnaire enclosed a cover letter outlining the overall purpose and contents of the study. Participants’ anonymity and confidentiality were emphasized, in accordance with the guidelines for personal data processing defined by the Italian privacy law (Legislative Decree no. 101 of August 10, 2018). The letter also specified that participation was voluntary, and participants were entitled to withdraw at any time without any requirement to justify their decision.

More than half of the sample consisted of women (52.9%) and the most frequent work sectors were health, social services, and law enforcement (26.2%). Most participants were technicians (e.g., computer technician, nurse) (31.8%), held a high school degree (46.6%), worked with a full-time open-ended contract (57.6%), and had a mean age of 41.57 years (*SD* = 10.51). On average, participants’ job tenure was equal to 9.65 years (*SD* = 8.50) and their mean contractual working hours per week were 34.51 h (*SD* = 8.24), while they declared to actually work 37.34 h (*SD* = 9.46).

### 2.3. Strategy of Analysis

#### 2.3.1. Exploratory Factor Analysis

The structure of the core dimensions (BAT-C) of burnout as well as the secondary dimensions (BAT-S) was explored using a principal component analysis (PCA) with varimax rotation in SPSS 23. Bartlett’s test of sphericity and the Keiser–Meyer–Olkin (KMO) measure were applied as a measure of sampling adequacy. The sample was considered adequate if the KMO value was higher than 0.70 and Bartlett’s test was significant (*p* < 0.001). As a criterion, factors reporting an eigenvalue ≥1 were retained. In addition, item loadings are considered satisfactory when greater than 0.50 [53].

#### 2.3.2. Internal Consistency

The scale reliability for the general BAT-C and BAT-S measures and their subscale scores were estimated using Cronbach’s alpha coefficient. As a rule of thumb, values exceeding 0.70 provide evidence of an adequate scale reliability [54]. The item-total correlations were also calculated to evaluate whether the items actually measured different facets of burnout core and secondary symptoms. The cut-off score for acceptable item-total correlations was set to be between ≥0.30 and ≤0.70 to ensure the coherence between each item and the whole scale, but also to avoid redundant and unnecessary items [55].

#### 2.3.3. Confirmatory Factor Analysis

A confirmatory factor analysis was performed on the 33 BAT items using the AMOS software [56]. To assess model fit, different fit indices were used: the χ^2^ goodness-of-fit statistic; the root mean square error of approximation (RMSEA), the standardized root mean square residual (SRMR), the Tucker–Lewis index (TLI) and the comparative fit index (CFI). Values lower than 0.08 for SRMR and RMSEA and higher than 0.90 for CFI and TLI indicated an acceptable fit to the data [57,58].

#### 2.3.4. Convergent and Discriminant Validity

The convergent and discriminant validity of the BAT was assessed through the comparison of four alternative MTMM models [59]. For reasons of clarity, these models were identified with numbers ranging from 11 to 14. Among the BAT scales, only the BAT-C was included in the analyses. This forced choice was made because the alternative burnout measure included (i.e., the MBI-GS) did not assess secondary symptoms of burnout. Furthermore, the current study is focused on the MBI dimensions of exhaustion and cynicism, which are considered as the core components of the construct, both in theoretical terms [60] and according to empirical evidence [42,61].

First, the correlated traits–correlated methods model (CT-CM), here named as Model 11, was assessed as the target model against which three alternative CFA models were compared. In the first model, the correlation among all traits corresponding to the burnout dimensions included in the BAT-C and MBI-GS (i.e., the latent factors of exhaustion, cynicism/mental distance, cognitive impairment, emotional impairment) were allowed to vary (i.e., the latent BAT-C and MBI-GS). In contrast, trait and method factors were not allowed to correlate with one another.

In the second step, a no traits–correlated method model (NT-CM) was tested (i.e., Model 12). In contrast to the CT-CM model, the NT-CM model assumed that the structure of the data was better explained by the corresponding method (i.e., burnout instrument), thus trait factors were not specified. By comparing a model where latent burnout dimensions or traits are specified (i.e., Model 11), with a model where these six factors are not specified (i.e., Model 12), we explored to what extent the latent measures (i.e., BAT-C and MBI-GS) were correlated. Thus, the comparison between these two models provides support for the convergent validity of the scales included, as independent but correlated measures of the same construct.

In Model 13, the perfectly correlated traits-correlated methods (PCT-CM) model, the traits (i.e., latent burnout dimensions) were perfectly correlated (i.e., equal to 1) and the methods (i.e., BAT-C and MBI-GS) were freely correlated with each other. By comparing a model where traits are free to correlate (Model 11) with a model where perfect correlations are specified (Model 13), we explored the extent to which the burnout dimensions included as latent traits were distinguishable from each other. Hence, a significant difference between Model 11 and Model 13 would suggest that trait factors are not collinear and are actually tapping different burnout dimensions. Hence, this would provide evidence of discriminate validity among traits.

Model 14, the correlated traits-perfectly correlated methods (CT-PCM) model, was equivalent to Model 11, except the correlations between the latent measure factors (i.e., BAT-C and MBI-GS) were constrained to 1.0. A significant difference between Model 11 and Model 14 denotes the discriminant validity of measures (i.e., BAT-C and MBI-GS). To compare MTMM models, the Chi-square difference test and change in CFI were examined [57]. Accordingly, alternative models were considered as substantially different when Δχ^2^ was significant at the *p* < 0.01 level and ΔCFI was greater than 0.01 [62].

#### 2.3.5. Predictive and Incremental Validity Analysis

Beyond the ability to explain or predict variance, it is essential that the burnout conceptualization and measure embraced in the BAT-C explains unique or incremental criterion variance not accounted for by the conceptually related and established measure of the MBI-GS. Thus, the predictive and incremental validity of the BAT-C when controlling for the MBI-GS, was examined. Subsequently, we performed the same analysis while reversing the entering order of the instruments measuring burnout core symptoms. By first entering the BAT and then the MBI-GS, we also appreciated the added value of the MBI-GS above and beyond the BAT.

Accordingly, we conducted two hierarchical regression analyses including BAT-S as a criterion variable, to estimate which measure of burnout core components concurrently predicted burnout secondary symptoms, and we entered sex and age as covariates in the first step of the analysis. The following steps of the hierarchical regression diverged between the two alternative models. In the first model, the second step included MBI-GS scores and in the third step, BAT-C was entered.

In the second solution, the entering order was reversed: BAT-C was entered in the second step and MBI-GS scores were included in the last step of analysis.

In doing so, we assessed the effect of the second independent variable, considering the covariates, the first predictor and the correlation between the two predictors (i.e., MBI-GS and BAT-C).

#### 2.3.6. Cross-National Comparison

As the BAT research consortium includes a network of academic researchers from across the world, the current paper also provides a breakdown of the burnout levels from seven nationally representative samples [48]. This supplementary analysis allows for the comparison of the descriptive results obtained on the Italian sample with participants across seven nationally representative samples.

## 3. Results

### 3.1. Exploratory Factor Analysis

Concerning the BAT-C, Bartlett’s test of sphericity was significant, with χ^2^ = 9842.33, df = 253, *p* < 0.001. The KMO measure was equal to 0.95, considered a highly satisfactory value [63]. These results suggest that correlations between BAT-C items were adequate to conduct a PCA. As shown in Table 2, the results of the PCA suggest that four factors with eigenvalues above 1 produced a four-component rotated solution for the 23 BAT-C items, which explained 64.84% of the total variance. After direct varimax orthogonal rotating, the structural matrix showed that the first component explained 20.49% of the variance and comprised eight items corresponding to the exhaustion dimension of the original BAT-C scale (loadings between 0.53 and 0.77). The second factor explained 15.21% of the variance and comprised five items corresponding to the cognitive impairment dimension (loadings between 0.63 and 0.81). The third factor accounted for 15.60% of the variance and included five items corresponding to the mental distance dimension (loadings between 0.67 and 0.80). The fourth factor explained 13.54% of the variance and involved five items referring to the emotional impairment component (loadings between 0.64 and 0.76).

Preliminary analyses on the BAT-S items revealed a significant Bartlett’s test of sphericity (χ^2^ = 2757.84, df = 45, *p* < 0.001) and a satisfactory KMO measure (0.91). These results concur in indicating that the data were suitable for factor analysis. Table 3 reports PCA results concerning the 10 BAT-S items. A two-factor rotated solution explained 56.86% of the common variance. The first factor included five items corresponding to the psychological complaints original scale and explained 31.62% of the variance, with loadings ranging from 0.62 to 0.79. Five items originally comprised in the psychosomatic complaints scale loaded on a second factor with values ranging between 0.50 and 0.75, and explained 25.24% of the total variance.

### 3.2. Internal Consistency

Cronbach’s alpha values and corrected item-total correlation coefficients are reported in Table 2 and Table 3. The BAT-C reported a Cronbach’s alpha coefficient of 0.94, and this value ranged from 0.85 to 0.90 for the four subscales.

The internal consistency of the total BAT-S scale resulted in a Cronbach’s alpha coefficient of 0.87, whereas psychological complaints and psychosomatic complaints reported an alpha value of 0.82 and 0.78, respectively.

Examination of the corrected item-total correlation coefficients indicated that all items substantially contributed to measure a core common construct, with values ranging between 0.52 and 0.70 for BAT-C and between 0.49 and 0.71 for BAT-S.

### 3.3. Confirmatory Factor Analysis

Table 4 reports the results of comparisons between four alternative models aimed to validate the measurement structure of the scale. In the first model (M1), all items loaded on a general latent BAT-J factor. This model assumed that burnout represents a syndrome embracing a broad range of symptoms relying on a single psychological condition (i.e., job burnout).

The second model (M2) was a two-factor model with the 23 BAT-C items and the 10 BAT-S items loading on the corresponding latent variable. According to this model, burnout is better conceived as the combination of two specific facets, consisting of the primary and secondary symptoms of this syndrome. Next, we evaluated a third model (M3), assuming six distinct but correlated factors (i.e., exhaustion, mental distance, impaired cognitive control, impaired emotional control, psychological complaints, and psychosomatic complaints). This model assumed that burnout is better described as the result of six facets corresponding to its main symptom categories.

In line with the conceptualization of burnout as a set of symptoms clustered in between core and secondary dimensions, the fourth model (M4) was a second-order model with four first-order factors (i.e., exhaustion, mental distance, cognitive impairment, emotional impairment) loading on a core symptoms higher-order factor (BAT-C), while the remaining two factors (psychological complaints and psychosomatic complaints) are captured by a second general factor corresponding to secondary symptoms (BAT-S). Our first model (M1) reported a relatively poor fit, especially in relation to CFI = 0.72 and TLI = 0.71. In addition, SRMR was unsatisfactory with a value equal to 0.09, thus, above the described cut-off point. The bi-factor model (M2) did not provide a fully satisfactory fit given that the improvement of the SRMR value, equal to 0.07, was still associated with CFI and TLI indices corresponding to 0.76 and 0.75, respectively, thus below the acceptance criteria. The third model (M3) fit the data significantly better than M1 and M2, with all indices meeting the corresponding thresholds: χ^2^ (480) = 1292.88, *p* < 0.001; CFI = 0.93; TLI = 0.93; SRMR = 0.04; RMSEA = 0.05. This result suggests the suitability of a solution which differentiates between six clusters of burnout symptoms. The last model (M4) reported a similar fit to the data, with insignificant changes in all the inspected indices. 

Factor loadings for M4 are reported in Table 5, with loadings ranging between λ = 0.62 (*p* < 0.001) and λ = 0.87 (*p* < 0.001) for the 23 items composing BAT-C first-order factors (i.e., exhaustion, mental distance, cognitive impairment, emotional impairment) and between λ = 0.57 (*p* < 0.001) and λ = 0.83 (*p* < 0.001) for the 10 items loading on the BAT-S first-order factors (i.e., psychological complaints, psychosomatic complaints).

Furthermore, loadings on the second-order factor BAT-C were λ = 0.88 (*p* < 0.001) for exhaustion, λ = 0.76 (*p* < 0.001) for mental distance, λ = 0.75 (*p* < 0.001) for cognitive impairment and λ = 0.82 (*p* < 0.001) for emotional impairment. Loadings on the second-order factor BAT-S corresponded to λ = 0.97 (*p* < 0.001) for psychological complaints and λ = 0.86 (*p* < 0.001) for psychosomatic complaints. Therefore, the second-order CFA solution was accepted as a reasonable model for the data.

### 3.4. Convergent and Discriminant Validity

**Figure 1:**
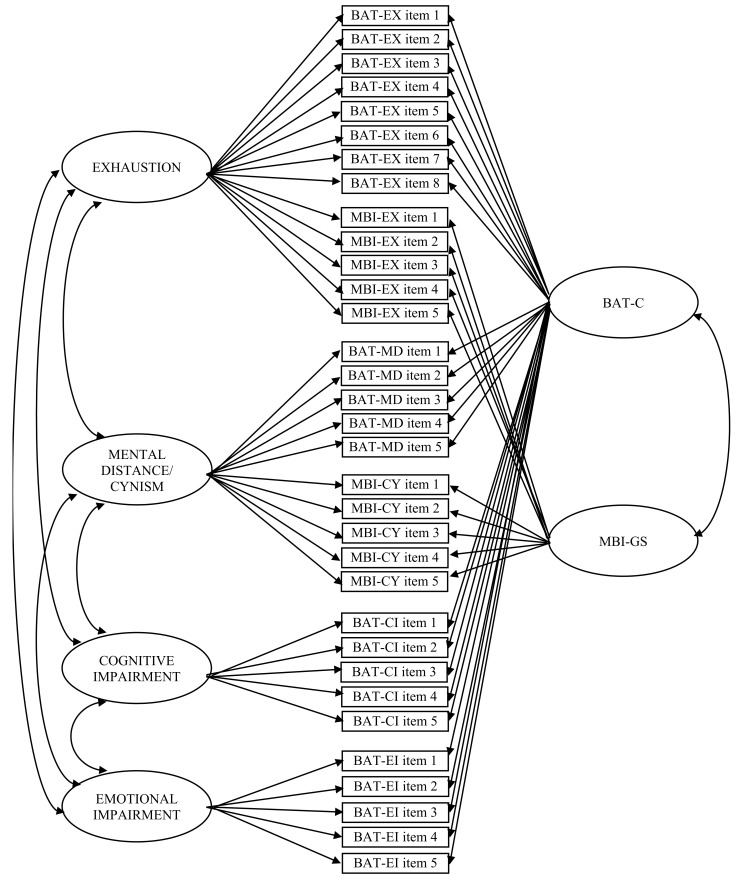
Correlated traits–correlated methods (CT-CM) model for the Burnout Assessment Tool—Core (BAT-C) and the Maslach Burnout Inventory-General Survey (MBI-GS). *Note.* EX = Exhaustion; MD = Mental Distance; CY = Cynicism; CI = Cognitive Impairment; EI = Emotional Impairment.

A summary of MTMM model comparisons is displayed in Table 6. In the CT-CM model, here defined as Model 11 (Figure 1), all items loaded significantly on the trait and method factors, except for the third item of the emotional impairment scale. Model 11 reported the best overall fit, with the lowest χ^2^ to degrees of freedom ratio (equal to 2.75), the highest CFI (0.95) and TLI (0.94), and the lowest SRMR (0.03) and RMSEA values (0.04). The latent correlation between trait factors were all significant (*p* < 0.05), with values ranging from 0.21 to 0.39. In particular, the exhaustion trait reported a correlation equal to r = 0.23 (*p* = 0.013) with the mental distance/cynicism trait, r = 0.36 (*p* < 0.001) with the cognitive impairment trait, and r = 0.39 (*p* < 0.001) with the emotional impairment trait. The mental distance/cynicism trait displayed a correlation of r = 0.21 (*p* = 0.008) with cognitive impairment and r = 0.24 (*p* = 0.006) with emotional impairment. Between the traits of cognitive and emotional impairment the correlation reported a value of r = 0.39 (*p* < 0.001). The measure factors (i.e., BAT-C and MBI-GS) reported a correlation equal to r = 0.89 (*p* < 0.001).

By comparing Model 11 with Model 12, we assessed for evidence of convergent validity of the BAT-C and MBI-GS scales. The χ^2^ difference was significant (Δχ^2^ (39) = 3211.58, *p* < 0.0001) and the difference in practical fit was substantial, with the NT-CM model (Model 12) reporting the worst fit to the data, suggesting that the independent measures of job burnout (BAT-C and MBI-GS) are correlated. 

We assessed for evidence of discriminant validity of burnout dimensions by comparing the CT-CM model (Model 11) with the PCT-CM model (Model 13). The χ^2^ difference was significant (Δχ^2^ (6) = 404.87, *p* < 0.0001). The fit indices of Model 13 were poorer than Model 11, with ΔCFI = 0.03 and SRMR = 0.11. These results provided evidence that burnout dimensions should be distinguished from each other.

Next, the discrimination of the methods (i.e., BAT-C and MBI-GS) was assessed through the comparison of Model 11 and Model 14 (the CT-PCM model). The comparison between a model where measure factors were free to correlate (i.e., Model 11) and a model assuming a perfect correlation between them (i.e., Model 14) was conducted in order to substantiate the discriminant validity between measures. The χ^2^ difference was significant (Δχ^2^ (1) = 54.92, *p* < 0.0001) and the difference in CFI fit index was significant ΔCFI = 0.01. Moreover, in Model 14, two items showed insignificant loadings to the latent method factor. They were the third and fourth items of the BAT-C mental distance scale (i.e., “I feel a strong aversion towards my job”; “I feel indifferent about my job”). This result corroborated the hypothesized independence between the BAT-C and the MBI-GS.

### 3.5. Predictive and Incremental Validity Analysis

As previously described, the predictive and incremental validity of the BAT above and beyond was assessed through the estimation of two alternative models.

As reported in Table 7, in the first model we entered the MBI-GS (second step) and the BAT-C (third step). The MBI-GS accounted for 41% (*p* < 0.001) of the variance. The core burnout symptoms assessed through the BAT-C added an additional 8% (*p* < 0.001) of the unique variance in the criterion variable.

The second solution was based on the reversed order of predictors entry. The second step added the BAT-C scores, which accounted for 48% (*p* < 0.001) of the variance. As highlighted in the third step, the additional variance in burnout secondary symptoms explained by entering the MBI-GS scores was equal to 1% (*p* < 0.001).

These findings provide evidence for the added value of job burnout core components, as defined and operationalized in the BAT-C, in predicting burnout secondary symptoms (i.e., psychological and psychosomatic complaints). On the other hand, 1% of variance in burnout symptoms is explained by MBI-GS when controlling for the BAT-C.

### 3.6. Cross-National Comparison

Table 8 reports the descriptive results of the burnout core symptoms assessed through the BAT-C in Italy and seven other nationally representative samples. According to this table, the Italian sample (*n* = 738) reported a higher mean score in burnout core symptoms—assessed through the BAT-C—than samples from Finland, Austria, Germany, and The Netherlands. On the other hand, Italian employees participating in the current study reported a lower mean value in comparison to samples from Belgium, Ireland, and Japan, with the latter reporting the largest difference.

## 4. Discussion

The purpose of our study was to evaluate—for the first time—the psychometric properties of the Italian version of the Burnout Assessment Tool (BAT), an instrument grounded in the conceptualization of burnout proposed by Schaufeli et al. [46] with the intent of addressing the shortcomings of the leading measures of burnout, most notably the MBI (for an overview, see [44]). The exploratory and confirmatory factor analyses provided evidence for the hypothesized 4-factor structure (i.e., exhaustion, mental distance, cognitive and emotional impairment) concerning the core symptoms of burnout. With respect to the secondary symptoms of burnout, both exploratory and confirmatory analyses corroborate the validity of the hypothesized 2-factor structure (i.e., psychological and psychosomatic symptoms) with item saturations on the appropriate factor. These results substantiate the theoretical and unique conceptualization of burnout underlying the development of the BAT [36], able to distinguish core and secondary burnout symptoms [48].

Furthermore, the obtained results support the empirical consistency of a second-order model with four first-order factors (i.e., exhaustion, mental distance, cognitive impairment, emotional impairment) loading on a core symptoms higher-order factor (BAT-C). This finding suggests that in the Italian context, the BAT can be understood as a measure of a genuine burnout syndrome. This issue constitutes the main deviation from the current most widely used burnout instrument, the MBI, where its scales are conceived as separate and not combinable dimensions [6]. While both measures operationalize burnout as a multidimensional construct, the BAT only recognizes four core symptoms as being closely interrelated and expressive of a unique underlying condition of burnout. According to our results, the reliability of all subscales of the BAT-C and BAT- S is highly satisfactory. In addition, the core symptoms of burnout (i.e., BAT-C) reported an optimal Cronbach’s alpha coefficient (0.94), thus confirming the high internal consistency of the items for all of the scales considered [48,49,50,51].

To investigate construct validity, an MTMM was performed to explore the relationship between the BAT-C and MBI-GS. The comparison between the different models attested the discriminant validity between the burnout dimensions (here clustered as exhaustion, mental distance/cynicism, cognitive impairment, emotional impairment) and the convergent validity between the same dimensions of the MBI, as well as the relative independence between the two instruments. Our findings, in line with previous studies [49], confirm the assumption that the BAT-C provides an effective measure of burnout as illustrated by the convergence of burnout symptoms (i.e., traits) also measured by the MBI-GS. On the other hand, it is also clear that no total overlap exists between the two methods, or questionnaires, considered here. In other words, from an empirical standpoint, the BAT should be considered a novel, alternative burnout instrument that is not redundant with existing burnout instruments, but rather adds a specific contribution to the assessment of its core symptoms, which specifically include both emotional and cognitive impairment, in addition to the evaluation of secondary symptoms [46].

This evidence is additionally reinforced by results on predictive and incremental validity. In particular, our results attested that burnout core symptoms, included in the BAT-C, significantly predicted burnout secondary symptoms, over and beyond what was explained by the MBI-GS.

Finally, regarding the comparison among Italian data and the seven countries included in the cross-national study [48], our sample presented relatively higher burnout scores as compared to Finland, Austria, Germany, and The Netherlands. As widely established in other countries [49,50,51], the empirical evidence provided in the current study strongly concurs in proposing that even in the Italian context, the BAT may offer both a conceptually robust and empirically reliable tool for measuring burnout in work settings.

### 4.1. Study Limitations

The main limitation of the study lies in the sample size, which is also not nationally representative of the Italian working population. Even though we collected a heterogeneous sample, which included a variety of sectors and professional roles, it was not possible to collect a representative sample. This aspect may represent a drawback, especially in defining Italian norms and to perform a cross-national comparison with other countries that collected representative samples [4,48].

Another possible issue related to sample characteristics refers to its non-problematic nature, since our sample comprised mainly healthy workers. In order to check the discriminative power of the BAT and the possibility of identifying groups and individuals with different risk levels for burnout (low, moderate, and high), future studies should also include burnout patients. Having a single burnout score, as in the case of the BAT, is very helpful for distinguishing between healthy employees and employees who lie on the spectrum between being at risk for burnout and experiencing early symptoms of severe burnout. However, this distinction requires clinically validated cut-off scores, which are not available in Italy, as is the case in most of the other countries [36,64]. Therefore, future studies should combine BAT self-reported data with medical interviews in order to define specific cut-off scores for burnout risk among Italian employees.

Additionally, our study only focused on psychometric properties related to reliability and factorial validity and the relationship between BAT and MBI. Future research should further examine construct validity, exploring, for example, the relationship between the BAT and other constructs, such as work characteristics, namely job demands and job resources, and individual characteristics, such as personality or personal resources. Criterion validity should also be investigated in more detail. In fact, we only tested the role of the core burnout symptoms in predicting secondary symptoms. We did not use any independent criterion, such as an external objective indicator. Future studies are needed to explore the predictive validity of the BAT in relation to sickness absence data-records, performance outcomes and health indicators. Finally, future studies should also include depressive mood, included in the BAT conceptual model among the secondary symptoms, in order to further explore the relationship between burnout and depression.

### 4.2. Practical Implications

In line with international results on the BAT [48,49,50,51], this study showed that the BAT represents a reliable, valid, and free to use alternative to the MBI-GS in the Italian context. It is crucial to have a free to use tool that allows researchers to compare data from different countries, sectors, and professional roles. A sound burnout measure, able to provide an overall burnout score, such as the BAT, could be particularly relevant for psychosocial risk assessment and work-related organizational interventions. In fact, this tool could be used as a potential outcome of the work-related stress risk assessment, to identify the impact of organizational factors and work characteristics on workers’ well-being. In particular, a single burnout total score is very helpful to develop cut-off scores that could be used to assess burnout prevalence within group, organizations, and countries. Moreover, developing cut-off points could be crucial to identify employees who are at risk for burnout in order to target them with preventive measures, as well as for evaluating the effectiveness of burnout interventions on burned-out employees.

## 5. Conclusions

Our study provided initial and promising evidence for the psychometric properties of the Italian version of a newly developed tool to measure burnout, namely the Burnout Assessment Tool, which intends to overcome some of the conceptual, methodological and practical limitations of the MBI. This tool measures core and secondary burnout symptoms and has already been validated in European and non-European countries. Our results demonstrated the high reliability and factorial validity of the Italian version of the BAT, as well as its construct validity in relation to the MBI. Moreover, the BAT predicted burnout secondary symptoms over and beyond the MBI. Accordingly, the BAT may represent a sound alternative to the MBI to measure burnout, also providing a burnout total score in addition to single dimension scores. This feature allows for having a comprehensive score of the syndrome, which could be of particular importance for practical purposes, such as assessing burnout, planning, and evaluating burnout interventions.

## Figures and Tables

**Table 1:** Description of study participants.

	Total Sample (*n =* 738)
***Gender***	
Female	52.9%
Male	47.1%
***Age***	
Mean (SD)	41.57 (*SD* = 10.51)
***Work sector***	
Health, social services, law enforcement	26.2%
Business services (e.g., consulting or ICT)	24.4%
Industry	7.9%
Public Administration	6.9%
Education sector	6.4%
Wholesale or retail trade, repairs	3.8%
Construction	2.2%
Tourism, hospitality, and catering	2.2%
Other	20.2%
***Work role***	
Technician (e.g., computer technician, nurse)	31.8%
White-collar workers (e.g., office clerk, secretary, salesperson)	30.6%
Professional (e.g., physician, teacher, lawyer, consultant)	18.6%
Manager (e.g., Manager, Supervisor, CEO)	8.3%
Blue-collar workers (e.g., cleaners, construction worker)	5.4%
Craftsman (e.g., electrician, plumber, blacksmith)	1.1%
Other	4.2%
***Educational level***	
Middle School	4.5%
High School	46.6%
University degree	40%
Post-graduate degree	8.9%
***Work contract***	
Full time open-ended contract	57.6%
Part time open-ended contract	23%
Full time fixed term contract	6.5%
Part time fixed term contract	1.8%
Other	11.1%
***Job tenure (years)***	
Mean (*SD*)	9.65 (SD = 8.50)
***Working hours by contract***	
Mean (*SD*)	34.51 (SD = 8.24)
***Effective working hours***	
Mean (*SD*)	37.34 (SD = 9.46)

**Table 2:** Exploratory factor analysis results of the BAT Core Symptoms (BAT-C).

Items				Factor Loadings			
	*M*	*SD*	r_tot_	Exhaustion	Mental Distance	Cognitive Impairment	Emotional Impairment
Al lavoro mi sento mentalmente esausto/a.	2.75	0.94	0.64	0.70			
2. Ogni cosa che faccio al lavoro mi richiede un grande sforzo.	2.52	1	0.61	0.72			
3. Dopo una giornata di lavoro, per me è difficile recuperare le energie.	2.60	1.02	0.60	0.77			
4. Al lavoro mi sento fisicamente esausto/a.	2.39	1.03	0.65	0.73			
5. La mattina, quando mi alzo, mi mancano le energie per cominciare una nuova giornata di lavoro.	2.37	1.06	0.67	0.71			
6. Vorrei essere più attivo/a sul lavoro, ma per qualche ragione non ci riesco.	2.24	1.06	0.67	0.53			
7. Se faccio uno sforzo sul lavoro, mi stanco più velocemente del consueto.	2.12	1	0.65	0.67			
8. Alla fine della mia giornata lavorativa, mi sento mentalmente esausto/a e svuotato/a.	2.62	1.04	0.67	0.73			
9. Ho difficoltà a provare un qualche entusiasmo per il mio lavoro.	2.14	1.05	0.61		0.63		
10. Al lavoro non penso molto a quello che faccio e agisco in modo meccanico.	1.78	0.97	0.55		0.70		
11. Provo una forte avversione per il mio lavoro.	1.85	1.05	0.70		0.76		
12. Mi sento indifferente rispetto al mio lavoro.	1.78	1.02	0.63		0.81		
13. Sono scettico/a rispetto al significato che il mio lavoro ha per gli altri.	2.07	1.12	0.62		0.67		
14. Al lavoro faccio fatica a mantenere l’attenzione.	2.00	0.85	0.62			0.73	
15. Quando lavoro ho difficoltà a pensare con lucidità	1.80	0.80	0.65			0.75	
16. Sul lavoro sono distratto/a e ho difficoltà a tenere a mente le cose.	1.92	0.81	0.58			0.79	
17. Quando lavoro faccio fatica a concentrarmi.	1.96	0.83	0.67			0.80	
18. Al lavoro faccio degli errori perché penso ad altro.	1.94	0.81	0.57			0.67	
19. Al lavoro mi sento incapace di controllare le mie emozioni.	1.86	0.87	0.52				0.76
20. Sul lavoro ho delle reazioni emotive che non mi appartengono	1.77	0.91	0.65				0.69
21. Mentre lavoro divento irritabile se le cose non vanno come vorrei.	2.13	0.96	0.48				0.76
22. Al lavoro mi capita di arrabbiarmi o sentirmi triste senza sapere perché.	1.76	0.93	0.66				0.64
23. Al lavoro mi capita di avere delle reazioni esagerate senza volerlo.	1.80	0.93	0.64				0.67
**Eigenvalue**				**4.71**	**3.50**	**3.59**	**3.11**
**% of variance**				**20.49**	**15.21**	**15.60**	**13.54**
**Cronbach’s α**				**0.90**	**0.87**	**0.89**	**0.85**

Note. r_tot_ = corrected item-total correlation.

**Table 3:** Exploratory factor analysis results of the BAT Secondary Symptoms (BAT-S).

Items				Factor Loadings	
	*M*	*SD*	r_tot_	Psychological Complaints	Psychosomatic Complaints
Faccio fatica ad addormentarmi o a mantenere il sonno.	2.29	1.15	0.60	0.67	
2. Tendo a preoccuparmi.	2.81	1.01	0.65	0.79	
3. Mi sento teso/a e stressato/a.	2.79	1	0.71	0.79	
4. Mi sento ansioso/a e/o soffro di attacchi di panico.	1.81	1.01	0.66	0.73	
5. Il rumore e la folla mi disturbano.	2.27	1.09	0.51	0.62	
6. Soffro di palpitazioni o dolori al petto.	1.61	0.92	0.61		0.50
7. Soffro di mal di stomaco e/o disturbi intestinali.	2.16	1.13	0.60		0.66
8. Soffro di mal di testa.	2.24	1.02	0.55		0.69
9. Soffro di dolori muscolari, ad esempio al collo, alle spalle o alla schiena.	2.72	1.13	0.59		0.68
10. Tendo ad ammalarmi facilmente.	1.68	0.86	0.49		0.75
**Eigenvalue**				**3.16**	**2.52**
**% of variance**				**31.62**	**25.24**
**Cronbach’s α**				**0.82**	**0.78**

Note. r_tot_ = corrected item-total correlations.

**Table 4:** Goodness of fit of alternative BAT models.

Model	χ^2^	*P*	df	CFI	TLI	SRMR	RMSEA [90%CI]
**M1.** Unidimensional model	6465.46	0.000	860	0.72	0.71	0.07	0.09 [0.09–0.10]
**M2**. Correlated four-factor model	3624.17	0.000	494	0.76	0.75	0.07	0.09 [0.09–0.10]
**M3.** Correlated six-factor model	1292.88	0.000	480	0.93	0.93	0.04	0.05 [0.04–0.05]
**M4.** Second-order model (6 first-order; 2 s-order)	1386.37	0.000	488	0.93	0.93	0.04	0.05 [0.04–0.05]
	Δχ^2^		Δdf		*p*		
M2 vs. M1	2841.29		366		<0.0001		
M3 vs. M1 M3 vs. M2	5172.58 2331.29		380 14		<0.0001 <0.0001		
M4 vs. M1 M4 vs. M2 M4 vs. M3	5079.09 2331.29 93.49		372 6 8		<0.0001 <0.0001 <0.0001		

Note. CFI = Comparative Fit Index; TLI = Tucker-Lewis index; SRMR = Standardized Root Mean Square Residual; RMSEA = Root Mean Square Error of Approximation.

**Table 5:** Factor loadings of M1 with six first-order factors and two second-order factors (*n =* 738).

First-Order Factors					
BAT-C			BAT-S		
First-Order Factor	Item	λ	First-Order Factor	Item	λ
Exhaustion	1	0.73 ***	Psychological Complaints	1	0.65 ***
	2	0.71 ***		2	0.74 ***
	3	0.73 ***		3	0.83 ***
	4	0.76 ***		4	0.73 ***
	5	0.76 ***		5	0.57 ***
	6	0.69 ***			
	7	0.74 ***	Psychosomatic Complaints	1	0.66 ***
	8	0.77 ***		2	0.67 ***
				3	0.63 ***
Mental Distance	1	0.71 ***		4	0.67 ***
	2	0.66 ***		5	0.57 ***
	3	0.86 ***			
	4	0.81 ***			
	5	0.72 ***			
					
Cognitive Impairment	1	0.78 ***			
	2	0.82 ***			
	3	0.78 ***			
	4	0.87 ***			
	5	0.69 ***			
					
Emotional Impairment	1	0.66 ***			
	2	0.81 ***			
	3	0.62 ***			
	4	0.79 ***			
	5	0.79 ***			
**Second-order factors**					
BAT-C		γ	BAT-S		γ
Exhaustion		0.88 ***	Psychological Complaints		0.97 ***
Mental Distance		0.76 ***	Psychosomatic Complaints		0.86 ***
Cognitive Impairment		0.75 ***			
Emotional Impairment		0.82 ***			
**Correlation between second-order factors**					
BAT-C ↔ BAT-S		0.89 ***			

Note. *** *p* < 0.001.

**Table 6:** Model fit indices for the MTMM models with BAT-C and MBI-GS.

Model	χ^2^	*P*	df	CFI	TLI	SRMR	RMSEA [90%CI]
**M11.** CT-CM model	1251.50	0.000	455	0.95	0.94	0.03	0.04 [0.04–0.05]
**M12.** NT-CM model	4463.08	0.000	494	0.76	0.74	0.07	0.10 [0.10–0.10]
**M13.** PCT-CM model	1656.37	0.000	461	0.92	0.91	0.11	0.05 [0.05–0.06]
**M14.** CT-PCM model	1306.42	0.000	456	0.94	0.94	0.08	0.05 [0.04–0.06]
	Δχ^2^		*p*		Δdf		
M12 vs. M11	3211.58		<0.0001		39		
M13 vs. M11	404.87		<0.0001		6		
M14 vs. M11	54.92		<0.0001		1		

Note. CT-CM = Correlated Traits/Correlated Methods; NT-CM = No Traits/Correlated Methods; PCT-CM = Perfectly Correlated Traits/Correlated Methods; CT-PCM = Correlated Traits/Perfectly Correlated Methods. CFI = Comparative Fit Index; TLI = Tucker–Lewis index; SRMR = Standardized Root Mean Square Residual; RMSEA = Root Mean Square Error of Approximation.

**Table 7:** Hierarchical multiple regression predicting burnout secondary symptoms (BAT-S).

	*R* ^2^	*F*	*β*	*p*	Δ*R*^2^
*Step 1*: Covariate 1. Sex 2. Age	0.09	37.89	−0.31 0.05	0.000 0.170	0.09
MBI-GS and BAT-C					
*Step 2*: MBI-GS	0.50	248.00	0.65	0.000	0.41
*Step 3*: BAT-C	0.58	253.03	0.53	0.000	0.08
Alternative solution: BAT-C and MBI-GS					
*Step 3*: BAT-C	0.57	803.38	0.71	0.000	0.48
*Step 3*: MBI-GS	0.58	22.23	0.22	0.000	0.01

**Table 8:** Descriptive statistics of mean scores in burnout core symptoms assessed through the BAT-C across 8 countries.

Burnout Core Symptoms (BAT-C)						
	Mean	SD	Median	25th Percentile	50th Percentile	75th Percentile
Italy (*n =* 738)	2.09	0.64	2.04	1.61	2.04	2.48
The Netherlands (*n =* 1500)	2.05	0.63	2	1.59	2	2.38
Belgium (Flanders) (*n =* 1500)	2.19	0.83	2.05	1.53	2.05	2.80
Germany (*n =* 1073)	2.08	0.70	2	1.55	2	2.49
Austria (*n =* 1059)	2.05	0.72	1.93	1.55	1.93	2.43
Japan (*n =* 1032)	2.51	0.80	2.46	1.98	2.46	3
Finland (*n =* 2299)	2.04	0.54	2	1.67	2	2.35
Ireland (*n =* 431)	2.41	0.64	2.01	1.60	2.01	2.51

## Data Availability

The data that support the findings of this study are available from the corresponding author, C.C. upon reasonable request.

## Appendix A

**Table A1:** Italian version of the Burnout Assessment Tool (BAT)—Core Symptoms.

	Mai	Raramente	Qualche Volta	Spesso	Sempre
Esaurimento					
Al lavoro mi sento mentalmente esausto/a	☐	☐	☐	☐	☐
Ogni cosa che faccio al lavoro mi richiede un grande sforzo	☐	☐	☐	☐	☐
Dopo una giornata di lavoro, per me è difficile recuperare le energie	☐	☐	☐	☐	☐
Al lavoro mi sento fisicamente esausto/a	☐	☐	☐	☐	☐
La mattina, quando mi alzo, mi mancano le energie per cominciare una nuova giornata di lavoro	☐	☐	☐	☐	☐
Vorrei essere più attivo/a sul lavoro, ma per qualche ragione non ci riesco	☐	☐	☐	☐	☐
Se faccio uno sforzo sul lavoro, mi stanco più velocemente del consueto	☐	☐	☐	☐	☐
Alla fine della mia giornata lavorativa, mi sento mentalmente esausto/a e svuotato/a	☐	☐	☐	☐	☐
**Distanza mentale**					
Ho difficoltà a provare un qualche entusiasmo per il mio lavoro	☐	☐	☐	☐	☐
Al lavoro non penso molto a quello che faccio e agisco in modo meccanico	☐	☐	☐	☐	☐
Provo una forte avversione per il mio lavoro	☐	☐	☐	☐	☐
Mi sento indifferente rispetto al mio lavoro	☐	☐	☐	☐	☐
Sono scettico/a rispetto al significato che il mio lavoro ha per gli altri	☐	☐	☐	☐	☐
**Perdita di controllo cognitivo**					
Al lavoro faccio fatica a mantenere l’attenzione	☐	☐	☐	☐	☐
Quando lavoro ho difficoltà a pensare con lucidità	☐	☐	☐	☐	☐
Sul lavoro sono distratto/a e ho difficoltà a tenere a mente le cose	☐	☐	☐	☐	☐
Quando lavoro faccio fatica a concentrarmi	☐	☐	☐	☐	☐
Al lavoro faccio degli errori perché penso ad altro	☐	☐	☐	☐	☐
**Perdita di controllo emotivo**					
Al lavoro mi sento incapace di controllare le mie emozioni	☐	☐	☐	☐	☐
Sul lavoro ho delle reazioni emotive che non mi appartengono	☐	☐	☐	☐	☐
Mentre lavoro divento irritabile se le cose non vanno come vorrei	☐	☐	☐	☐	☐
Al lavoro mi capita di arrabbiarmi o sentirmi triste senza sapere perché	☐	☐	☐	☐	☐
Al lavoro mi capita di avere delle reazioni esagerate senza volerlo	☐	☐	☐	☐	☐

**Table A2:** Italian version of the Burnout Assessment Tool (BAT)-Secondary symptoms.

	Mai	Raramente	Qualche Volta	Spesso	Sempre
Disturbi psicologici					
Faccio fatica ad addormentarmi o a mantenere il sonno	☐	☐	☐	☐	☐
Tendo a preoccuparmi	☐	☐	☐	☐	☐
Mi sento teso/a e stressato/a	☐	☐	☐	☐	☐
Mi sento ansioso/a e/o soffro di attacchi di panico	☐	☐	☐	☐	☐
Il rumore e la folla mi disturbano	☐	☐	☐	☐	☐
**Disturbi psicosomatici**					
Soffro di palpitazioni o di dolori al petto	☐	☐	☐	☐	☐
Soffro di disturbi di stomaco e/o disturbi intestinali	☐	☐	☐	☐	☐
Soffro di mal di testa	☐	☐	☐	☐	☐
Soffro di dolori muscolari, ad esempio al collo, alle spalle o alla schiena	☐	☐	☐	☐	☐
Tendo ad ammalarmi facilmente	☐	☐	☐	☐	☐
